# New Role, New Country: introducing US physician assistants to Scotland

**DOI:** 10.1186/1478-4491-5-13

**Published:** 2007-05-04

**Authors:** James Buchan, Fiona O'May, Jane Ball

**Affiliations:** 1 Queen Margaret University, Clerwood Terrace, Edinburgh EH12 8TS, UK; 2 Employment Research, Hove, East Sussex, BN3 5DQ, UK

## Abstract

This paper draws from research commissioned by the Scottish Executive Health Department (SEHD). It provides a case study in the introduction of a new health care worker role into an already well established and "mature" workforce configuration It assesses the role of US style physician assistants (PAs), as a precursor to planned "piloting" of the PA role within the National Health Service (NHS) in Scotland.

The evidence base for the use of PAs is examined, and ways in which an established role in one health system (the USA) could be introduced to another country, where the role is "new" and unfamiliar, are explored.

The history of the development of the PA role in the US also highlights a sometimes somewhat problematic relationship between P nursing profession. The paper highlights that the concept of the PA role as a 'dependent practitioner' is not well understood or developed in the NHS, where autonomous practice within regulated professions is the norm. In the PA model, responsibility is shared, but accountability rests with the supervising physician. Clarity of role definition, and engendering mutual respect based on fair treatment and effective management of multi-disciplinary teams will be pre-requisites for effective deployment of this new role in the NHS in Scotland.

## Background

This paper draws from research commissioned by the Scottish Executive Health Department (SEHD). It assesses the role of US style physician assistants (PAs), as a precursor to planned "piloting" of the PA role within the National Health Service (NHS) in Scotland. As such, it reports on the evidence base for the use of PAs, and then discusses how an established role in one health system (the USA) could be introduced to another country, where the role was "new" and unfamiliar. For a comprehensive assessment and description of the development of the PA role in the USA, see the core text by Hooker and Cawley [1].

The primary focus of the paper is to identify lessons for consideration during planning and implementation of the planned "pilot", when a small number of US trained and based PAs were to be recruited to work in the NHS in Scotland for a period of up to two years. The recruitment phase was completed in autumn 2006, with the PAs beginning work in the NHS. The pilot phase will be subject to independent evaluation, and the expectation is that, if successful, the training of PAs will then be introduced in Scotland.

The paper reports key findings from the literature review, and follow up findings, and focuses on key messages for policy makers. As such, the paper provides a case study in the policy and practice implications of introducing a new type of health worker role into an already well established and "mature" workforce context.

This literature review reports on publications from the USA. Whilst the physician assistant role, or similar, does exist in other countries, such as Canada, England, the Netherlands, Taiwan, and India, the role originated in, and is best established and most fully examined in the US context.

The literature review was based on a search which was conducted using the following key terms: physician(s) assistant, PA, utilisation, deployment, employment, impact, costs, cost benefit, evaluation, working relationship, role differentiation, role delegation, substitution. The time period searched was 1990 – 2005, and English language only. Databases searched were: Medline, CINAHL, BNI, PsycInfo and Web of Science. Websites consulted included The American Academy of Physician Assistants (AAPA), and the National Library of Medicine (NLM). In total, approximately 160 references were identified.

This brief review of US publications covers the following key areas:

• background overview and demographics;

• education;

• role descriptions and assessments; and

• costs, benefits and evaluation of impact.

### Overview of the US PA workforce

The physician assistant (PA) is a widely recognised, fully integrated licensed health provider working within the US health care system. The role has been in existence for about forty years, and was developed both in response to a shortage of doctors in primary care in the 1960s and in an attempt to increase access to health care for people in underserved, particularly rural, areas.

The first physician assistant trainees in the US were military paramedics – many were returnees from the Vietnam war. Formal education was established in 1965 and the Duke University Physician Assistant programme produced the first graduates in 1967. Duke University was also instrumental in the early development of regulatory legislation for the profession, initially within North Carolina [2]. From these early initiatives there was growth in the number of PA educational programmes, numbering 138 in 2006 [3].

Historically, the majority of physician assistants have worked in primary care, but increasing numbers are now working in hospitals, satellite clinics, community practice and government agencies. Less than half of all PAs now work in primary care [4]. PAs work in a wide variety of healthcare settings, including occupational health, forensic medicine, radiology, cardiothoracic surgery, cardiology, respiratory medicine, gastroenterology, general medicine, obstetrics and gynaecology, paediatrics, anaesthesia, cancer surgery, emergency medicine, dermatology, rheumatology, health promotion, geriatrics, care and nursing homes, organ procurement, psychiatry and neonatal care [5].

The number of PAs currently in the US is around 66000, with a predicted growth to 90000 by 2010 [3]. Although the physician assistant profession has historically been predominantly male, women now comprise over 60% of PAs working in the US [6]. This can be in part be explained by the fact that many of the initial cohorts of PAs, who were men, have now reached or are nearing retirement age. The flexibility of the job is reportedly attractive to women who have childcare responsibilities. Many PAs have been recruited from other healthcare jobs, often as a second career [7], but recently, there has been an increasing trend towards applications from younger life science graduates, who are embarking on a first career.

Recent debate in the US has focused on the likely extent of the projected physician shortage [8,9], which has been highlighted as one reason for continued growth in PA employment [10].

Employment as a PA appears to be an attractive career option in the US. A recent survey of PAs indicated that 89% said they would do the same job over again (cited in [3]). LaBarbera [11] mailed a survey to 2323 PAs, randomly selected from the AAPA mailing list, in an attempt to identify the factors that PAs feel contribute to vocational satisfaction. The survey achieved a 50% response rate, and was found to be representative in composition. Overall, PAs were found to be highly satisfied with their careers (92.4%), specialty choices (90.6%) and jobs (81.8%), and 87% said they were highly or fairly likely to recommend a PA as a career.

### Education

PA education is modelled on physician training. PAs are taught in programmes often co-located within medical schools and teaching hospitals. PA students frequently share classes, facilities and clinical rotations with medical students. As previously mentioned, the number of PA programmes has grown rapidly across the USA. Applicants to PA programmes must complete at least two years of college courses in basic science and behavioural science as prerequisites to PA training, which is analogous to the pre-medicine studies required for medical students.

The average education programme length has increased over the years, and was reported recently to be 26 months [12]. This programme is broad-based and generalist. The first year comprises basic medical science courses after which students enter the clinical phase of training. This includes classroom instruction and participatory clinical rotations in medical and surgical specialties – family medicine; internal medicine; obstetrics and gynaecology; paediatrics; general surgery; emergency medicine, psychiatry and elective sub-specialties. In order to graduate, students have to complete 2000 hours of supervised clinical practice, and pass a national standardised exam. They then have to apply for licensure in whichever State they are practising. After graduation, the PAs continue learning at work and through continuing medical education (CME), for which they must log 100 hours every two years (half of which must be courses/educational events approved by the AAPA). In addition, they must sit a recertification exam every six years, in order to remain licensed practitioners. The average size of a graduation class from a PA programme has increased from 25 to just over 35 students between 1993 and 2003, despite the fact that new programs tend to start with smaller classes [4].

The overall number of graduates emerging from PA programmes has consistently increased over the past 11 years, rising from 2000 in 1995, to more than 4500 in 2003, and is currently around 4700 per annum [10]. Similarly, the number of enrolments in PA programs has risen steadily [13].

### Role

The AAPA definition of the profession is

"*Physician assistants are health professionals licensed or, in the case of those employed by the federal government, credentialed, to practice medicine with physician supervision. Physician assistants are qualified by graduation from an accredited physician assistant educational program and/or certification by the National Commission on Certification of Physician Assistants. Within the physician/PA relationship, physician assistants exert autonomy in medical decision-making and provide a broad range of diagnostic and therapeutic services. The clinical role of physician assistants includes primary and specialty care in medical and surgical practice settings in rural and urban areas. Physician assistant practice is centered on patient care, and may include educational, research, and administrative activities" *[14].

Recent economic pressures have promoted the increased use of PAs and expanded their scope of practice. It is reported that the role of the PA can be broken down into four main components, whereby they:

• practise medicine with physician supervision

• exercise autonomy in medical decision making (always with a physician partner)

• provide a broad range of diagnostic and therapeutic services

• may also perform educational, research, and administrative activities [3].

PAs are always supervised by a physician, but in most cases this does not mean direct supervision, but may often be given remotely, via telephone or video, for example. "*PAs are not independent practitioners, but practice-focused autonomous professionals delivering care in partnership with physicians, in a role described as 'negotiated performance autonomy' *[[15], p.485], which takes into account differences between skills sets. This means they can staff satellite clinics, provide on-call services and deliver care in rural areas, as the physician partner does not need to be physically present for the PA to practise.

Several studies have looked at the differences between PA and other so called "mid level practitioner" roles (see e.g. [16], particularly the nurse practitioner (NP). A key difference cited is the independent licensure bestowed on NPs [17,18].

Hooker and Cipher [19] examined PA and NP prescribing, and reported differing prescriptive authority, with PAs licensed in 47 states, and NPs licensed in 40 states (although Cox [20] reported advanced nurse practitioners (ANPs) can prescribe in all 50 states). They found that PAs were more likely to prescribe a controlled substance than were physicians or NPs, except in rural areas, where NPs wrote more prescriptions, but that overall, both PAs and NPs prescribed in a manner similar to that of physicians. They also found that proportionally more primary care NPs and PAs than physicians were located in non-metropolitan areas.

### Evaluation of impact of PA

There is an evidence base of literature on PA "productivity" stretching back to the 1970s. Early work suggested that the substitution ratio of PAs and NPs for physicians was between 0.5 and 0.75, (meaning that one PA could "replace" one half to three quarters of a physician [21].

More recently, Larson et al. [22], in an analysis of productivity data (collected in 1993–1994) from a nationally representative sample of PAs, showed that they conducted 61.4 outpatient visits per week compared with 74.2 visits performed by physicians, for an overall physician full-time equivalent (FTE) estimate of 0.83. However, productivity of PAs varied markedly across practice specialty and location, with generalist PAs performing more visits than their specialist counterparts. Rural PA productivity was higher than urban productivity because of the concentration of generalist PAs in rural settings. The authors concluded that a generalist PA physician FTE estimate of 0.75 appears to be more accurate than the proposed measure of 0.5 under discussion at the time of the study.

In general, reported patient satisfaction with PAs has been high, whether in their own right, or whether compared with physicians and/or NPs [see e.g. [15,23,24]]. Roblin et al. [25] carried out a large-scale retrospective evaluation of patient satisfaction surveys (n = 41,209 patients) in the Atlanta metropolitan area. They looked at satisfaction in relation to practitioner type and across three scales; practitioner interaction, care access and overall experience. The main hypothesis was that the likelihood of patient satisfaction would not significantly differ between PAs or NPs and physicians attending a visit. The study however did not differentiate between PAs or NPs, presenting them both as midlevel practitioners. In the main, PAs/NPs represented in this study were more likely to attend visits for minor acute illness and physician visits for chronic disease. The main finding was that overall, as far as patients were concerned, the NP or PA does as good a job as the physician.

However, the authors concluded that there were other factors which had a more profound influence on patient satisfaction, including both time of visit, and length of time spent on visit. In addition, the primary care provided by PA/NPs was cost-saving, in terms of saving money while providing sustained or improved patient outcomes.

Miller et al. [26] carried out a literature review and conducted an internal record review to examine the use of PAs in the trauma centre of a large community hospital. Current and historical outcomes were analysed, including patient demographics, type of trauma, injury severity score, transfer times, and length of stay. In addition, fiscal data were examined. Questionnaires to elicit physician perceptions of resource savings were given to all eight trauma surgeons affiliated with the trauma department. Reported improvements related to the appointment and utilisation of PAs included: increased time savings of 4–5 hours per day, per physician; transfer time to operating room decreased by 43% and to intensive care unit by 51%; length of stay for admissions decreased by 13% and for neurotrauma intensive care patients, by 33%. Resource savings were reported by virtue of the fact that PAs could see outpatients in a clinical setting and perform bedside procedures without needing the supervising physician to be present. In addition, financial savings were also evident, as the cost of the PA trauma department can be offset by generated revenue charges. Decreased length of stay in critical care units and hospital resulted in significant savings also. This was a localised small scale study, but was felt to be a viable model of care provision for other trauma centres where it was not possible to maintain a surgical residency programme.

From her analysis of utilisation of PAs in the hospital setting, Duffy [27] suggests that the delegation of resident and house staff responsibilities to PAs will facilitate improved inpatient training experiences and more efficient physicians. Highlighted were administrative and non-educational tasks, which if delegated to non-physicians could considerably increase the value of the patient experience while reducing residents' hours.

Health workforce and policy analysts have been interested in the cost effectiveness of PAs since they were first introduced back in the 1960s. McKibbin [28] carried out an early review of cost effectiveness assessment of PAs, which concluded that the utilisation of PAs had a positive correlation with productivity measures and significant cost savings. However, he applied some caveats, as at that point, the generalisability of cost effectiveness had not been demonstrated, patients and third parties were not guaranteed to benefit, and reimbursement policies were inadequate and likely to impact on the cost-effective utilisation of PAs. A more recent review of the economic aspects of the PA role was carried out by Hooker [29], which suggested that the majority of economic research to date has focused on cost-effectiveness, using physicians or NPs for comparison. His findings with regard to the economics of PA practice were:

• A PA can perform at least 75% of a physician's tasks at a cost of 44% of the physician's salary (based on 1999 salary information);

• A PA can safely assume at least 83% of primary care visits without direct physician supervision;

• Cost-benefit analyses of PA-delivered primary care suggest the use of resources is less than physicians, under comparable circumstances

• The cost of training a PA is approximately one fifth that of a physician

• Owing to the difference in the length of education between PAs and physicians, the PA provides 5 years of patient care valued at $380,000 (1999 rates) before the physician completes a primary care residency, and enters health care practice.

He concluded that these factors, plus the compensation-to-production ratio (this compares the salary and benefit cost to employ a PA [compensation] with the revenue generated for their services) establish the PA as one of the most cost-effective health care clinicians from the employer's perspective [[29], p.51].

Another estimate of the economic benefit of PAs to family/general medicine practices was undertaken by Gryzbicki et al. [30]. They monitored the daily activities of a part-time (0.56 FTE) PA within one practitioner owned general practice, using observational data from site visits, and semi-structured interviews with the PA and the employer. They also reviewed office records, billing records and appointment logs, and data were compared for accuracy and validity with national statistics. The PA saw younger patients with more acute conditions than the physician, and saw more patients and for longer than the physician. Gryzbicki et al. [30] calculated that, compared with a practice employing a FTE physician, the annual profit of a practice employing a full time equivalent PA would be $52, 592. They also determined a same-task substitution ratio of 0.86, a compensation-to-production ratio of 0.36 and a gross financial productivity (adjusted to 1.0 FTE) of $112, 572.

A study by Hooker [31] examined the costs, from the employer's perspective, associated with employing PAs within a non-profit, prepaid group practice in Oregon and Washington State. He used an acute episode of care as the unit of analysis, and the dependent variable was the total visit cost by provider type. The study data comprised 12,782 medical office visits made by patients in 1998 for one of four diagnoses, and were shown to be representative of the larger population. A total of 305 different providers were identified for inclusion in the study. At the time of the study, the mean annual salary for a primary care PA was $54,400, and for a primary care physician it was $124,600. Hooker found that in every medical condition managed by PAs, the total episode cost was less than a similar episode managed by a physician, regardless of patient and department variables. Few differences emerged in the use of resources and the rate of return visits for a diagnosis between physicians and PAs. Whilst no actual figures were given, within the primary care setting, PAs appeared to be cost-effective from an employment perspective.

A later study by Hooker [32] analysed differences in administrative practices between physicians and PAs working in occupational and environment medicine. The study site was a for-profit health organisation in Texas specialising in occupational and industrial health care and injury treatment services. Retrospective secondary data were gathered from employee administrative files, patient encounter files and billing records. These data were held in a patient encounter database, which contained a case or episode of injury information for an individual, which was used for analysis. At the time of the study, the mean annual salary was $143,056 for a physician and $74,208 for a PA. The study found that on average, PAs worked proportionately more hours than physicians, on approximately half the salary. Patients seen by PAs were more likely to keep their return appointments than patients seen by physicians (which might reflect satisfaction with care), and given that charges were fixed regardless of provider, a higher rate of return visits to see the PA may be viewed as beneficial to the organisation. While their productivity to compensation ratio suggests they may be economical members of the health team from a labour standpoint, some of their cost-effectiveness may be negated by a higher referral rate than the physicians. Further research into the role of PAs working in this specialty was recommended.

Roblin et al. [33] found that primary care practices that used more PAs/NPs in care delivery realised lower practitioner labour costs per visit than practices that used less. They analysed four years of computerised data on approximately two million visits provided by 206 practitioners in two departments, adult medicine and paediatrics, between 1997 and 2000. Their goal was to estimate the savings in labour costs per primary care visit that might be realised from increased use of PAs and NPs. They found that although estimated labour cost savings per visit were very low, in terms of a few dollars, the net savings to a managed care organisation (MCO) are substantial.

One important point to note is that the results of any cost effectiveness or cost/benefit assessment in the US will be influenced by the type of payment system, and by the pay relativities between PA and any comparator group (e.g. physician or NP). The average PA salary in the US is reported to be approximately US $84,000 (median $81,000) [34], varying markedly by experience and by practice setting (PAs earn more in urban and hospital environments and less in rural areas). The results of any cost assessment, or scope for cost based substitution will be largely dependent on the comparative cost of the PA and the potential "substitute".

The PA role in the US is expanding, and the PA workforce has been growing. Recent growth has been fuelled by physician shortages, and the main areas of high growth are in hospital based care. The number of courses and number of graduates has been increasing; proportionately more graduates are now women, and there has been a growth in younger female applicants to the PA profession. Employment prospects in the US appear good, and salaries have been increasing (see [1]).

### PAs – key issues for consideration in Scotland

The previous section of the review has reported on the key findings from the US literature on the role of PAs. This section moves on to assess the implications of introducing a US PA type role to the NHS in Scotland.

### Rationale(s) for use of PAs

Like the USA, one of the main drivers for exploring PA roles in the NHS in Scotland has been concern about shortages of doctors. In primary care, difficulties with the recruitment and retention of GPs, particularly in deprived and/or rural areas, have been an issue [e.g. [35]]. There has also been an impact of reductions in availability of "doctors hours" as a result of the implementation of the European Working Time Directive (EWTD). The impact of EWTD on junior doctors' hours has led to an overall reduction in the medical workforce capacity in secondary care.

It is necessary to reflect on the factors that are identified as having caused shortages in the NHS in Scotland and contrast these with the US context. If the reasons for the shortages differ between the two countries, then the solutions to the problem may also differ – i.e. it cannot be assumed that introducing PAs will have the same impact on the NHS in Scotland as it has had in the USA.

Whilst the main driver for interest in the PAs role in the NHS has been the need to increase capacity in the face of medical workforce constraints, other factors have also been highlighted. The following have been reported as attractions of introducing PAs to the NHS in Scotland:

• they have a holistic/generalist perspective (as opposed to doctors who 'know more and more about less and less');

• because of its genericism, the PA role has the potential to bridge the divide between primary and secondary care;

• experience/stability of PAs (i.e. as opposed to transient junior doctors in training grades);

• PAs have flexibility – potential to work in variety of settings/specialties; and

• PAs have a broader recruitment pool- attract different range of people, who have skills to offer the NHS.

The early phase of discussion about the potential introduction of a PA-type role in Scotland and elsewhere in the NHS in the United Kingdom included reported rivalry between health professions [36-39]. Whilst the prospect of PAs being deployed in the NHS has been received enthusiastically by some [e.g. [40,41]], other commentators, particularly from the nursing profession, have raised concerns regarding working relationships, impact on patients and services, and cost implications [36] and role definition [37]. Some from within the nursing profession have questioned the need for an additional non-physician service provider, given the development of the nurse practitioner (NP) who can meet the service needs within a team that is consultant-led, has prescription privileges and can also practise independently. This debate mirrors that which took place in the US when PAs were being developed.

### Developing a strategic approach to the introduction of PAs

It is clear from the US, that even in that "market driven" healthcare system, an essential role was played by 'pump-priming' from the federal government in the 1970s, to support the development of the PA role and the educational support required to underpin development. The NHS Scotland proposals for PA recruitment has the potential benefit of "whole system" co-ordination, as the NHS throughout the country is covered by the same health sector planning and workforce policy framework and regulatory environment.

### Induction of PAs

PAs recruited from the US require an effective induction programme. The critical point is deciding when the induction period ends and "real" employment begins. It is likely that this will be best achieved by mutual agreement between PA and supervisor, and will require flexibility.

Preparation of other staff, through communication and training, both before and after PAs arrive, is equally important, if not more so. They need to be well informed about PAs: their role; activities they do and do not undertake; how the PA fits in to the team; and supervision requirements. Staff also need to have the opportunity to discuss how the role relates to their own, particularly if they are working in similar roles, such as NPs.

### Supervision & working relationships

PAs in the NHS in Scotland will have a named supervisor (a physician), with a named deputy (also a physician) to provide cover for holidays/absence. In secondary care, PAs will be part of the medical team or 'firm', with consultants as the named supervisor. In primary care, a GP will be the designated supervising physician.

Research on PAs working in England [42] highlighted that the US and UK understanding of 'supervision' differs, both in terms of the nature of supervision and amount of time. As dependent practitioners, the US PAs recognise their own limitations, and used supervision to deal with specific queries that relate to limits of their scope of practice. In contrast, the UK 'supervisors' had the expectation that the PAs would require more generalised and ongoing support and advice. In the US, PAs are often supervised by more than one physician (reportedly on average by five physicians at any time), rather than having a one-to-one model. This in part reflects a move towards teams in multi-practice acute care.

### Deployment/Scope of practice

One reported potential key strength of the PA role in the NHS is that it is generic, giving the PAs the ability to be deployed in a variety of settings, and compensating for the progressive specialisation of medical staff (and to some extent nursing staff also). Retaining generic skills means that staff are more transferable. Prescribing rights (prescriptive authority) for PAs in the NHS have been highlighted by many commentators as an essential requirement of PA role. Legislation will be required to enable this to happen. This is likely to take some time.

One critical issue related to the scope of practice of PAs has been identified by US commentators: physicians hiring a PA to work in a speciality outside their own area of competence/expertise, for example, a GP using a PA to work in advanced dermatology. To maintain the underlying principles of the PA as dependent practitioner, the PA must always work within the scope of practice of the supervising physician and know/recognise their limits.

### Regulation & public protection

Regulation is essential to the long-term establishment of the PA role, both in the US and the UK.

In the UK, protection of the public is at the forefront of new developments in the regulation of healthcare practitioners. One of the problems from a regulatory perspective is that the PA role is new and untested. There is a need to have the education, role, competences, and accountabilities fully developed in order to ensure that legislation and regulation supports the boundaries and goals of a new role. It is often a requirement that a voluntary register is set up as a pre-requisite to statutory regulation. Thus regulators consider that there is a lot of work to be done before regulation can be achieved, yet from a service perspective the lack of regulation is a concern and a major obstacle to introduction of new roles.

The need for regulation is not just related to the protection of the public, but from a service/workforce perspective regulation enables standardisation, so that new roles are defined and recognisable, and skills are transferable between employers. New roles require meaningful systems to ensure local developments can be linked to a national framework/understanding of these roles. Regulators have the task of trying to bring together the different local developments.

### Pay and rewards

In the USA PA salaries average approximately US$ 84,000 [34], although it may be double this for experienced PAs working in some hospital specialities, such as cardio-thoracic. This is about half the salary of a family practitioner, thus the cost of a PA is nearer that of a physician in family practice (where about 45% of PAs work) than in hospital where the salary differential is usually greater.

In introducing PAs in the NHS there are issues of pay parity and equal pay with other staff groups to consider. Unlike the US health care system, there is a national pay system within the NHS, underpinned by a single job evaluation framework. Initial calibration of the PA role on this system will set the pay rate for the pilot staff to be employed in Scotland.

## Conclusion

The literature reviewed in this paper has highlighted many of the benefits of the PA role – both perceived and researched. However, this literature is from the USA. Given the differences in the health systems, in the type of education and role, and in pay and funding differences, it is clear that not all the findings from these studies can be extrapolated to a Scottish context. In reviewing the potential use of PAs in the NHS in Scotland, it will be important to recognise and take account of contextual differences. Successful introduction of PAs will depend on good preparation and ensuring that all staff and stakeholders involved are well informed, and ideally are fully supportive of the project. Anticipating some of the potential problems and concerns they may have will enable better preparation.

The challenge of introducing a new role is felt keenly by those occupying current or "traditional" roles. Doctors in the NHS in Scotland may be uncomfortable about relinquishing control of activities previously within their sphere of control. The nature of a PA role as a 'dependent practitioner' is not well understood or developed in the NHS, where autonomous practice within regulated professions is the norm. In the PA model, responsibility is shared, but accountability rests with the supervising physician. The history of the development of the PA role in the US also highlights a sometimes somewhat problematic relationship between PAs and the nursing profession; most particularly in the role overlap with nurses in advanced practitioner posts and working as nurse practitioners, leading some to question why there is a need for a PA role. Clarity of role definition, and engendering mutual respect based on fair treatment and effective management of multi-disciplinary teams will be pre-requisites for effective deployment.

The final point to note is that the recruitment of US PAs to the NHS in Scotland is designed as the first stage in a process leading (if the pilot is evaluated as successful) to educating new PAs in Scotland and deploying them in established posts in defined roles in identified care environments where they can make a cost effective contribution to delivering patient care. As such, the evaluation of the pilot project has to look beyond the US individuals who will be in the first posts, and assess the roles they perform, the impact that these roles have, and the receptiveness of NHS health system in Scotland to sustaining the new role.

## Authors' contributions

J Bu directed the study, developed the methodology and drafted parts of the paper; J Ba contributed to study design and drafted parts of the paper; F O'M conducted the literature review and contributed to drafting.
